# Adenylate cyclase A amplification and functional diversification during *Polyspondylium pallidum* development

**DOI:** 10.1186/s13227-022-00203-7

**Published:** 2022-10-19

**Authors:** Yoshinori Kawabe, Pauline Schaap

**Affiliations:** School of Life Sciences, University of Dundee, Dundee, DD15EH UK

**Keywords:** Excitable systems, CAMP oscillations, Adenylate cyclase A, Coordinated cell migration, Cell aggregation, Morphogenetic movement, Dictyostelia

## Abstract

**Background:**

In *Dictyostelium discoideum* (*Ddis*), adenylate cyclase A (ACA) critically generates the cAMP oscillations that coordinate aggregation and morphogenesis. Unlike group 4 species like *Ddis*, other groups do not use extracellular cAMP to aggregate. However, deletion of cAMP receptors (cARs) or extracellular phosphodiesterase (PdsA) in *Polyspondylium pallidum* (*Ppal*, group 2) blocks fruiting body formation, suggesting that cAMP oscillations ancestrally control post-aggregative morphogenesis. In group 2, the *acaA* gene underwent several duplications. We deleted the three *Ppal aca* genes to identify roles for either gene and tested whether *Ppal* shows transient cAMP-induced cAMP accumulation, which underpins oscillatory cAMP signalling.

**Results:**

In contrast to *Ddis*, pre-aggregative *Ppal* cells did not produce a pulse of cAMP upon stimulation with the cAR agonist 2′H-cAMP, but acquired this ability after aggregation. Deletion of *Ppal aca1*, *aca2* and *aca3* yielded different phenotypes. *aca1ˉ* cells showed relatively thin stalks, *aca2ˉ* showed delayed secondary sorogen formation and *aca3ˉ* formed less aggregation centers. The *aca1ˉaca2ˉ* and *aca1ˉaca3ˉ* mutants combined individual defects, while *aca2ˉaca3ˉ* and *aca1ˉaca3ˉaca2ˉ* additionally showed > 24 h delay in aggregation, with only few aggregates with fragmenting streams being formed. The fragments developed into small fruiting bodies with stalk and spore cells. Aggregation was restored in *aca2ˉaca3ˉ* and *aca1ˉaca3ˉaca2ˉ* by 2.5 mM 8Br-cAMP, a membrane-permeant activator of cAMP-dependent protein kinase (PKA). Like *Ddis*, *Ppal* sorogens also express the adenylate cyclases ACR and ACG. We found that prior to aggregation, *Ddis acaˉ/ACG* cells produced a pulse of cAMP upon stimulation with 2′H-cAMP, indicating that cAMP oscillations may not be dependent on ACA alone.

**Conclusions:**

The three *Ppal* replicates of *acaA* perform different roles in stalk morphogenesis, secondary branch formation and aggregation, but act together to enable development by activating PKA. While even an *aca1ˉaca3ˉaca2ˉ* mutant still forms (some) fruiting bodies, suggesting little need for ACA-induced cAMP oscillations in this process, we found that ACG also mediated transient cAMP-induced cAMP accumulation. It, therefore, remains likely that post-aggregative *Ppal* morphogenesis is organized by cAMP oscillations, favouring a previously proposed model, where cAR-regulated cAMP hydrolysis rather than its synthesis dominates oscillatory behaviour.

**Supplementary Information:**

The online version contains supplementary material available at 10.1186/s13227-022-00203-7.

## Background

Developing organisms need to coordinate cell differentiation with the generation of form. While differentiation largely results from regulation of gene expression, form can be generated by coordinated cell movement, cell division or changes in cell shape, either of which can act alone or in combination with others [1]. *D. discoideum* (*Ddis*) amoebas survive starvation by aggregating to form a migrating sorogen or slug, which turns into a fruiting body, consisting of spores and stalk cells. The chemotactic cell movements that cause aggregation as well as slug and fruiting body morphogenesis are organized by pulses of cAMP that are initially secreted by the most food-deprived cells and propagate as waves through the population by cAMP-induced cAMP synthesis [2–4]. The oscillating centre becomes the organizing tip of aggregates, slugs and fruiting bodies.

Dictyostelia can be subdivided into four major groups, with *Ddis* residing in group 4. The other group 4 species also use cAMP as chemoattractant [5] and are likely to use cAMP pulses to coordinate morphogenesis as well. However, this is not clear for species in the other three groups, which use the dipeptide glorin and other compounds as chemoattractant for aggregation [5–9]. Nevertheless, deletion of either cARs or PdsA from the group 2 species *P. pallidum (Ppal)* disorganized post-aggregative morphogenesis [10–12], while *D. minutum* in group 3 showed cAMP-induced cAMP synthesis and oscillatory cell movement only after aggregation [13, 14]. This suggested that non-group 4 species use cAMP oscillations to coordinate morphogenesis in the slug and fruiting body stage while using other chemoattractants for aggregation.

To test this hypothesis we deleted the three *acaA* homologs from *Ppal* individually and in combination. While single knock-outs in *aca1*, *aca2* or *aca3* showed subtle defects in primary stalk, side-branch formation and aggregation, respectively, triple *aca1ˉaca3ˉaca2ˉ* cells were very delayed in aggregation, but still formed some small fruiting bodies after a long delay. We explored whether other dictyostelid adenylate cyclases could also participate in pulsatile cAMP signalling.

## Results

### Spatio-temporal expression patterns of *P. pallidum acaA* homologs

In *D. discoideum (Ddis), acaA* shows complex expression from different promoters. The promoter proximal to the coding sequence directs high expression at the slug tip, the central promoter directs low expression in the prespore region, while the most distal promoter directs high expression during aggregation [15]. *P. pallidum (Ppal)* has three *acaA* genes, *aca1*, *aca2* and *aca3* (Additional file 1: Fig. S1). Comparative transcriptomics shows that these and other *acaA* genes across taxon groups are upregulated after starvation, with group 4 *acaA* genes showing peak expression during aggregation. *Aca* genes are most highly expressed in stalk cells in groups 1–3, but in group 4 expression is highest in cup cells, which are unique to group 4 (Additional File 1: Fig. S1).

**Fig. 1:**
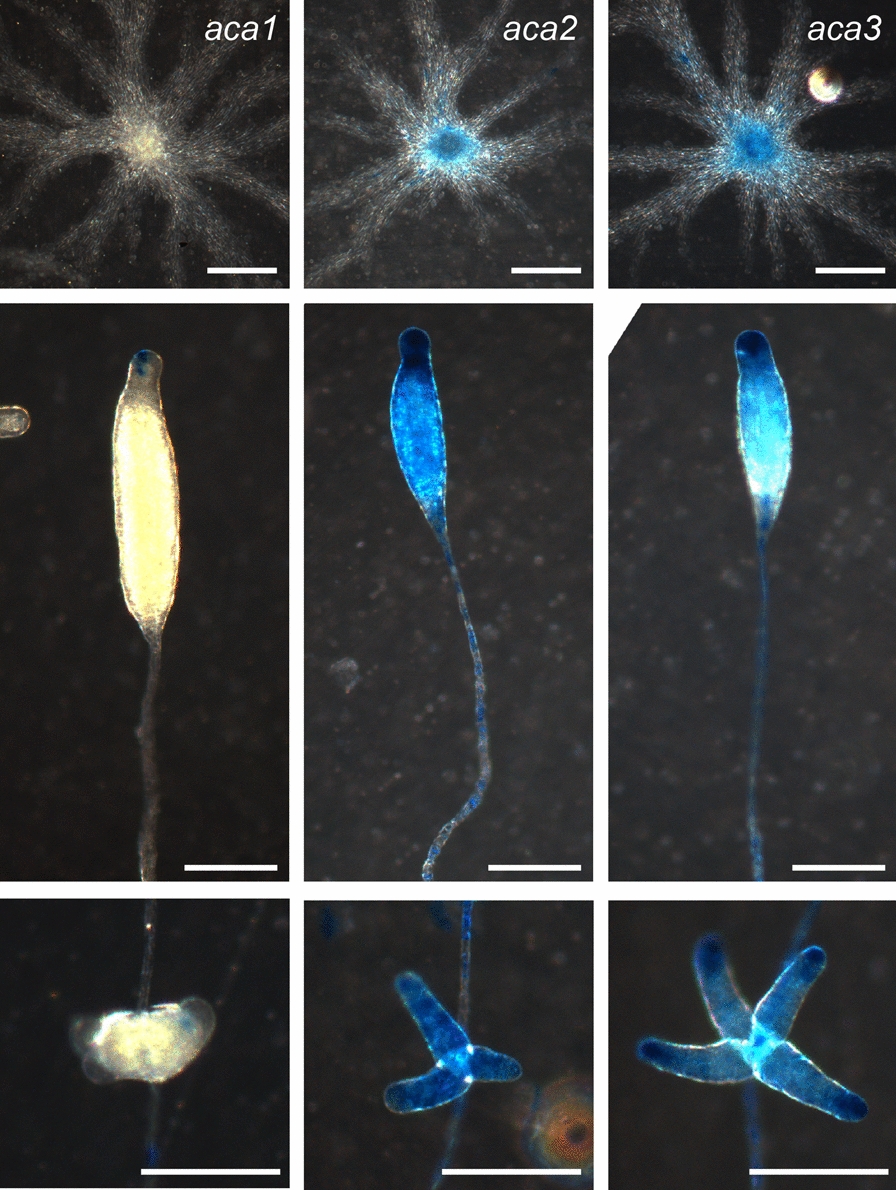
Expression patterns of *P. pallidum aca* genes. *Ppal* wild-type cells were transformed with gene fusions of the *LacZ* reporter and the intergenic regions upstream of the start-codon of the *Ppal aca1*, *aca2* and *aca3* genes. The cells were developed on NN agar until streaming aggregates (top), primary sorogens (centre) and secondary sorogens (bottom) had formed and intact structures were then fixed with glutaraldehyde and stained with X-gal. Bars: 0.1 mm

To investigate the spatial expression pattern of *Ppal aca1*, *aca2* and *aca3,* their promoter regions were fused to the *LacZ* reporter gene and transformed into *Ppal* cells. Developing structures were fixed and incubated with X-gal to visualize β-galactosidase activity. *Aca1* was not expressed during aggregation and started to be expressed weakly at the utmost tip region of the primary sorogen, and later sometimes in the tip of secondary sorogens (Fig. 1A). *Aca*2 and *aca*3 were already expressed in streaming aggregates and more strongly during post-aggregative development (Fig. 1B, C). In primary sorogens, *aca*2 was expressed throughout the structure but most strongly at the tip region. *Aca*3 expression was more specific to the tips of primary and secondary sorogens. Overall, the post-aggregative expression pattern of the three *Ppal aca*s resembles that of *Ddis acaA* with strongest expression at the sorogen tips [15, 16].

### Deletion of *aca genes* in *P. pallidum*

**Fig. 2:**
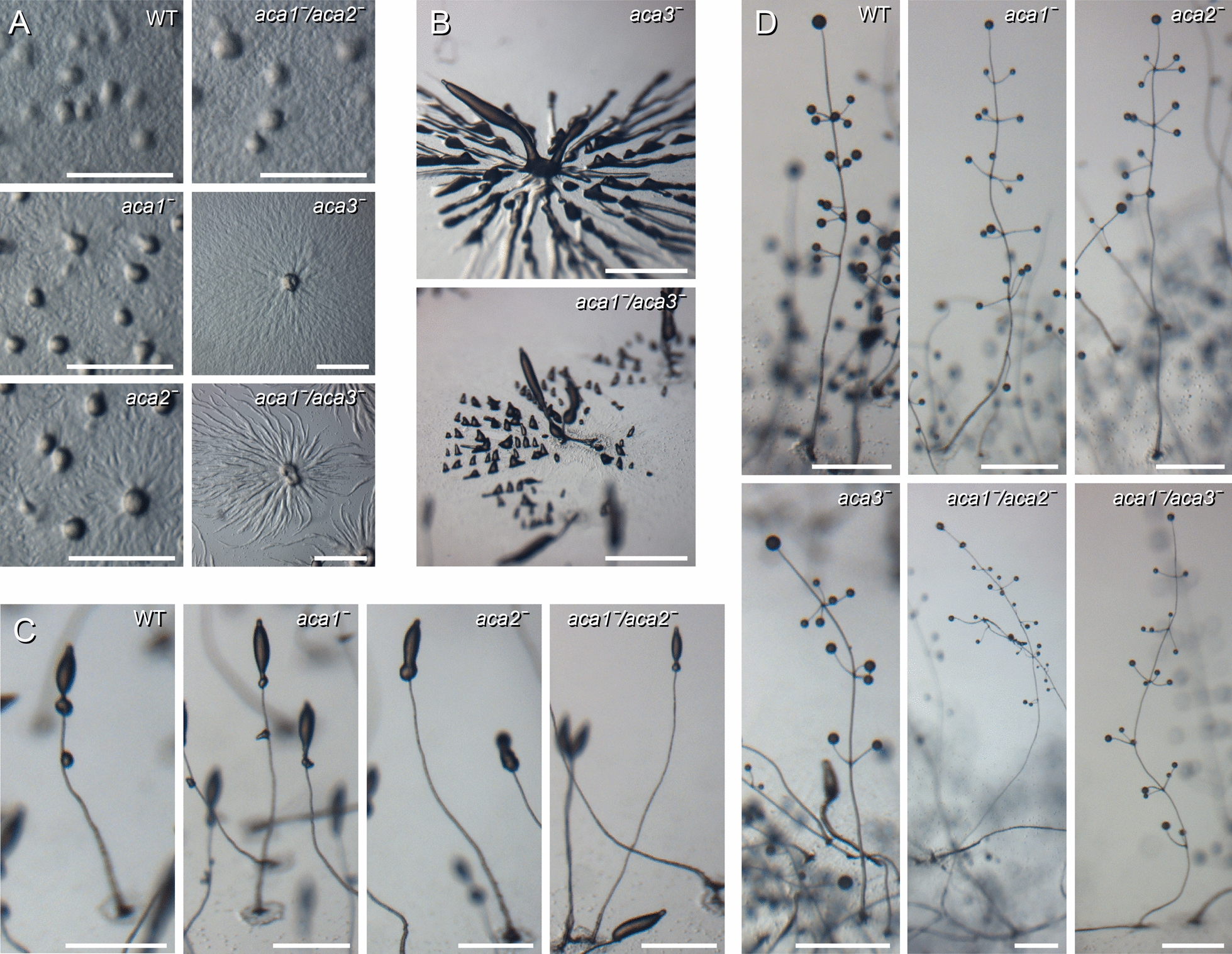
Development of single *aca*, *aca1ˉaca2ˉ* and *aca1ˉaca3ˉ* mutants. Wild type (WT) *Ppal* and *aca1ˉ, aca2ˉ, aca3ˉ, aca1ˉaca2ˉ* and *aca1ˉaca3ˉ* knock-outs were incubated at 22 °C on NN agar at 10^6^ cells/cm^2^. **A*** Aggregation:* the images show aggregates after 6 h or 8 h (*aca3ˉ* and *aca1ˉaca3ˉ*) of starvation. Bars: 1 mm. **B*** Tip formation:* aggregation streams of *aca3ˉ* and *aca1ˉaca3ˉ* forming tips at 20 h. Bars: 1.0 mm. **C*** Whorl mass separation*: *aca1ˉ, aca2ˉ* and WT at 20 h and *aca1ˉaca2ˉ* at 28 h of starvation*.* Bars: 0.5 mm. **D*** Fruiting bodies:* WT, *aca1ˉ,* and *aca2ˉ* had formed mature fruiting bodies at 28 h of starvation, while *aca3ˉ* and *aca1ˉaca3ˉ* took 32 h and *aca1ˉaca2ˉ* 48 h to complete their fruiting bodies. Bars: 0.5 mm

To assess the biological roles of the three *Ppal aca* genes, we replaced essential regions in each gene with the LoxP-NeoR cassette, in which *NeoR*, the single selectable marker of *Ppal* is flanked by *loxP* sites (Additional file 1: Fig. S2). The *aca1ˉ* clones aggregated normally and formed fruiting bodies with somewhat thinner and longer stalks than those of wild type *Ppal* (Fig. 2A, D). The *aca2ˉ* mutant aggregated and formed the primary sorogen normally but showed delayed formation of the first whorls of secondary sorogens (Fig. 2C). Such whorls arise at regular intervals when a posterior segment of the primary sorogen pinches off, while forming several regularly spaced tips, which each give rise to a small side branch. As a result, the branch-less lower stalk of *aca2ˉ* fruiting bodies was longer than in wild-type (Fig. 2D). The *aca3ˉ* mutant formed few aggregation centres with long streams (Fig. 2A), that partitioned into many tip-forming small aggregates that each gave rise to a small fruiting body. The central, large *aca3ˉ* aggregate produced a normal fruiting body (Fig. 2B, D). Overall, the phenotypes of single *aca* knock-out mutants were subtle. We tried to generate double and triple *aca* knock-outs by recycling the LoxP-NeoR cassette using the cre-recombinase expression vector pA15NLS.Cre [17]. This succeeded for the *aca1ˉ* mutant, allowing us to generate *aca1ˉaca2ˉ* and *aca1ˉaca3ˉ* double knock-outs, but not for the *aca2ˉ* or *aca3ˉ* knock-outs. The *aca1ˉaca3ˉ* phenotype combined features of *aca1ˉ* and *aca3ˉ* knock-out mutants. Similar to *aca3ˉ*, *aca1ˉaca3ˉ* formed few but large streaming aggregates (Fig. 2A, B), while the fruiting bodies showed thinner and longer stalks, like the *aca1ˉ* mutants (Fig. 2D). The *aca1ˉaca2ˉ* cells aggregated and formed primary sorogens normally. However, the separation of the first whorl only occurred after 28 h of starvation, when WT, *aca1ˉ*, and *aca2ˉ* had already formed fruiting bodies (Fig. 2C). As a result, *aca1ˉaca2ˉ* made very tall fruiting bodies with side branches only at the upper stalk (Fig. 2D).

The failure to recycle LoxP-NeoR cassette *of aca2ˉ* or *aca3ˉ* mutants was probably due to limited selectability of cells transformed with pA15NLS.Cre with its G418 selection cassette. We found that *Ppal* growth is also inhibited by the antibiotic Nourseothricin. This allowed us to use a Cre-recombinase expression vector pDM1483 [18] with a Nourseotricin selection cassette to eliminate LoxP-NeoR from *aca3ˉ* and *aca1ˉaca3ˉ* and to generate *aca3ˉaca2ˉ* and *aca1ˉaca3ˉaca2ˉ* knock-outs.

**Fig. 3:**
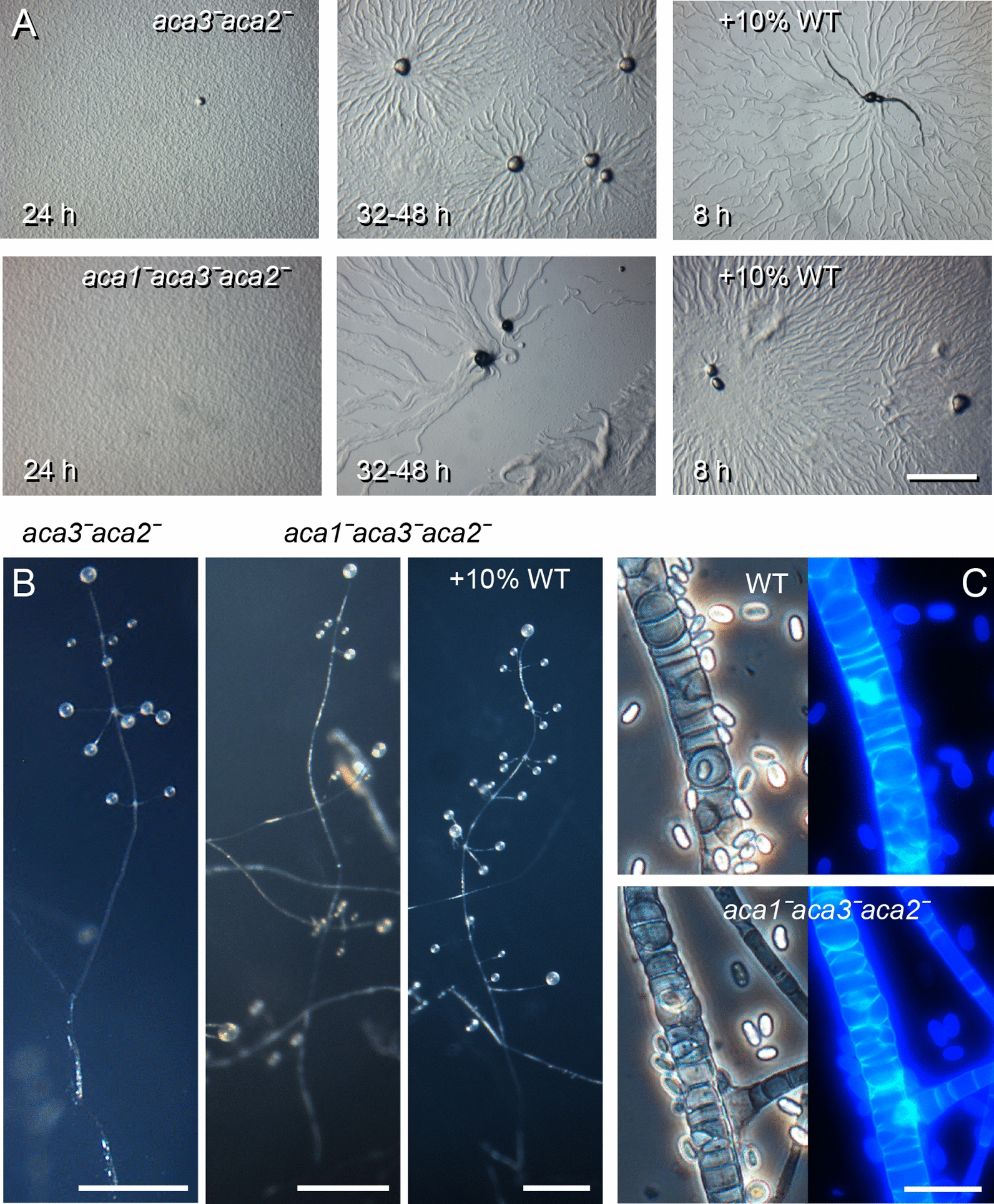
Development of *aca3ˉaca2ˉ* and *aca1ˉaca3ˉaca2ˉ mutants. ***A*** Aggregation: Ppal aca3ˉaca2ˉ* and *aca1ˉaca3ˉaca2ˉ* were starved on NN agar at 10^6^ cells/cm^2^ on their own or mixed with 10% wild-type *Ppal* for the indicated time periods. Bar: 1 mm. **B*** Fruiting bodies:* the cells were developed into fruiting bodies, which were imaged in situ*.* Bars: 0.5 mm. **C*** Spore and stalk cells: Ppal* WT and *aca1ˉaca3ˉaca2ˉ* fruiting bodies were transferred to 0.001% Calcofluor and imaged under phase contrast (left) and epifluorescence (right). Bar: 20 µm

Compared to wild-type, *aca1ˉaca2ˉ*, *aca1ˉaca3ˉ* and single *aca* knock-outs, which all initiated aggregation within 8 h of starvation, the *aca3ˉaca2ˉ* mutant only started to aggregate at 24 h or later (Fig. 3A). Only few aggregation foci were formed, which attracted very long aggregation streams. Starting from the initial (small) focus, mounds appeared at intervals within the streams, which each attracted downstream cells. Each of these mounds gave rise to a small, branched fruiting body, which, similar to *aca2ˉ,* showed a longer whorl-free lower stalk (Fig. 3B). The *aca1ˉaca3ˉaca2ˉ* phenotype combined features of the *aca1ˉaca2ˉ* and the *aca3ˉaca2ˉ* mutant. Similar to *aca3ˉaca2ˉ*, aggregation was much delayed with long streams appearing only after 24–48 h of starvation (Fig. 3A), which eventually broke up and gave rise to small fruiting bodies. These fruiting bodies showed delayed side-branch formation, like *aca1ˉaca2ˉ* (Fig. 3B). Staining of the *aca1ˉaca3ˉaca2ˉ* stalk and spore cells with the cellulose dye Calcofluor showed that it formed a normal primary and secondary stalk and elliptical spores encapsulated in cellulose walls (Fig. 3C), and this was also the case for all other *acaˉ* mutants (not shown).

To investigate whether the aggregation phenotypes of the *aca3ˉaca2ˉ* or *aca1ˉaca3ˉaca2ˉ* mutants were cell-autonomous, the mutants were developed as chimeras with wild-type cells. Introduction of 10% wild-type cells was sufficient to restore delayed aggregation of both mutants (Fig. 3A). The mixtures aggregated within 8 h of starvation-like wild-type cells, but still formed larger aggregation streams. In addition, the formation of secondary sorogens in *aca1ˉaca3ˉaca2ˉ* chimeras with wild-type was not as delayed as in *aca1ˉaca3ˉaca2ˉ* alone, resulting in formation of more normal fruiting bodies (Fig. 3B). These experiments show that the defects in aggregation and whorl separation of the *acaˉ* mutants are non-cell autonomous.

We also tested microcyst formation in *aca* knock-out mutants. Incubation with 0.2 M sorbitol for 24 h induced cyst formation in both wild type and *aca1ˉaca3ˉaca2ˉ* to the same degree (Additional File 1: Fig. S3), indicating that the *aca* genes are not required for encystation.

### Restoration of *aca1ˉaca3ˉaca2ˉ* aggregation by 8Br-cAMP.

**Fig. 4:**
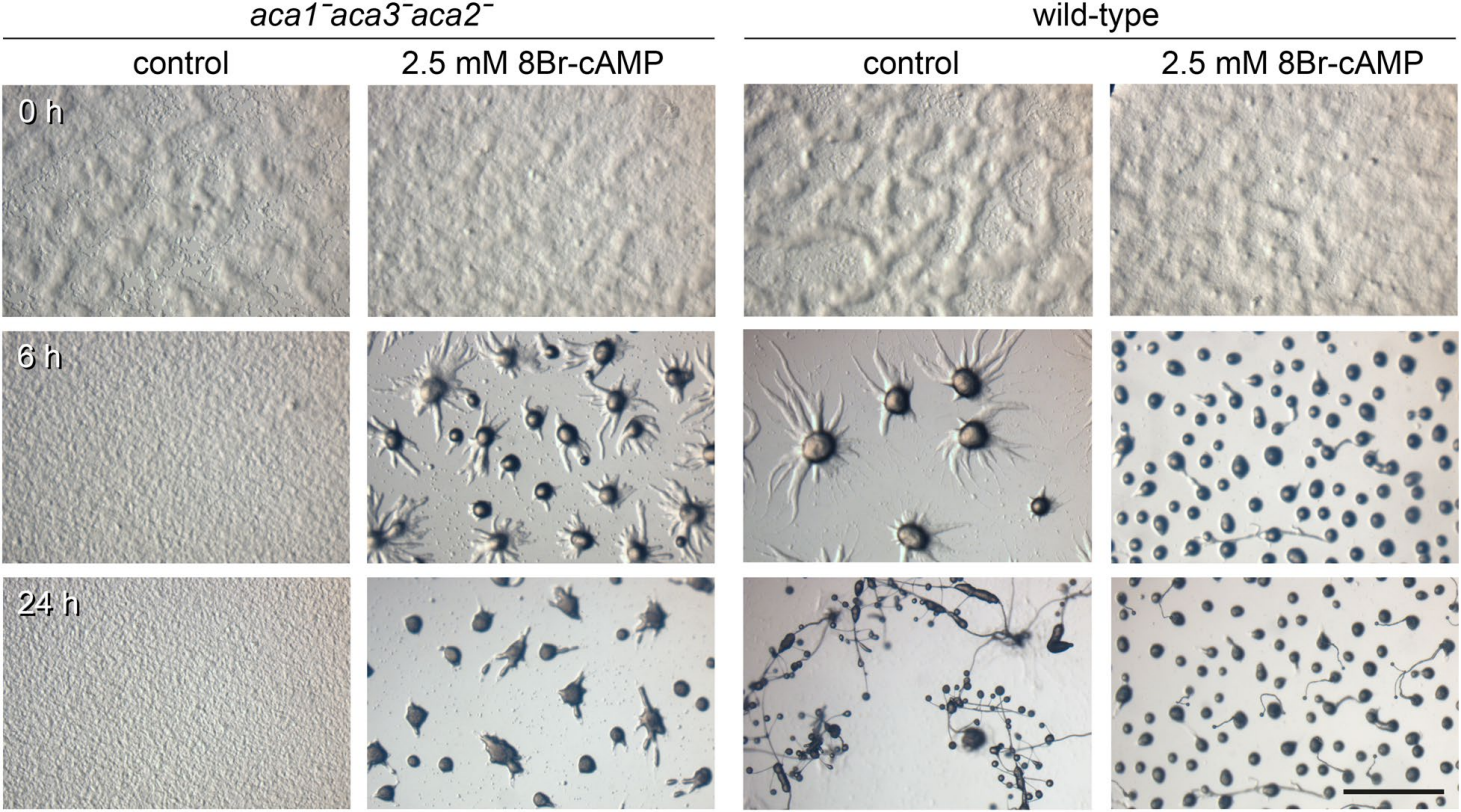
Effect of 8Br-cAMP on aggregation of *aca1ˉaca3ˉaca2ˉ* cells *Ppal aca1ˉaca3ˉaca2ˉ* and wild-type cells were incubated for 24 h on NN agar with and without 2.5 mM 8Br-cAMP and imaged in situ at the indicated timepoints. Bar: 1 mm

The strongly reduced initiation of aggregation centres and extensive delay in aggregation of both the *aca3ˉaca2ˉ* and *aca1ˉaca3ˉaca2ˉ* was unexpected, since *Ppal* does not use cAMP as attractant for aggregation, but most likely glorin [5, 9]. However, both *Ddis* and *Ppal* require PKA activity and, therefore, likely intracellular cAMP to develop competence for aggregation [19, 20]. To investigate whether lack of PKA activation due to the absence of intracellular cAMP cause the aggregation abnormalities in *aca3ˉaca2ˉ* and *aca1ˉaca3ˉaca2ˉ*, *aca1ˉaca3ˉaca2ˉ* cells were developed on agar containing 2.5 mM 8Br-cAMP, a membrane-permeant PKA agonist. While without 8Br-cAMP cells had not yet started to aggregate after 24 h of starvation, the 8Br-cAMP treated cells initiated many aggregation centres and almost completed aggregation within 6 h (Fig. 4). The aggregates remained, however, blocked in the mound stage and did not form fruiting bodies. This was, however, also the case for most *Ppal* WT aggregates developed on 8Br-cAMP agar. These results show that the *aca1ˉaca3ˉaca2ˉ* aggregation defect was likely caused by insufficient intracellular cAMP for PKA activation.

### cAMP relay in *Ppal* and in *Ddis aca-/ACG* cells

Despite the loss of all ACA activity, the *aca1ˉaca3ˉaca2ˉ* cells still made relatively normal fruiting bodies after a long delay. cAMP-induced excitation and adaptation of ACA underpins pulsatile cAMP signalling and wave propagation in *Ddis* [21, 22]*,* with cAMP receptors (cARs) and extracellular cAMP phosphodiesterase (PdsA) as essential components to, respectively, detect secreted cAMP and to hydrolyse it between pulses [23–25]. From earlier findings that *cAR* or *pdsA* null mutants in *Ppal* were specifically defective in fruiting body morphogenesis [11, 12], we concluded that cAMP waves mediated this process as they do in *Ddis* [4]. The present data imply that this is either not the case, or that the *aca1ˉaca3ˉaca2ˉ* cells have a means to compensate for loss of ACA activity.

**Fig. 5:**
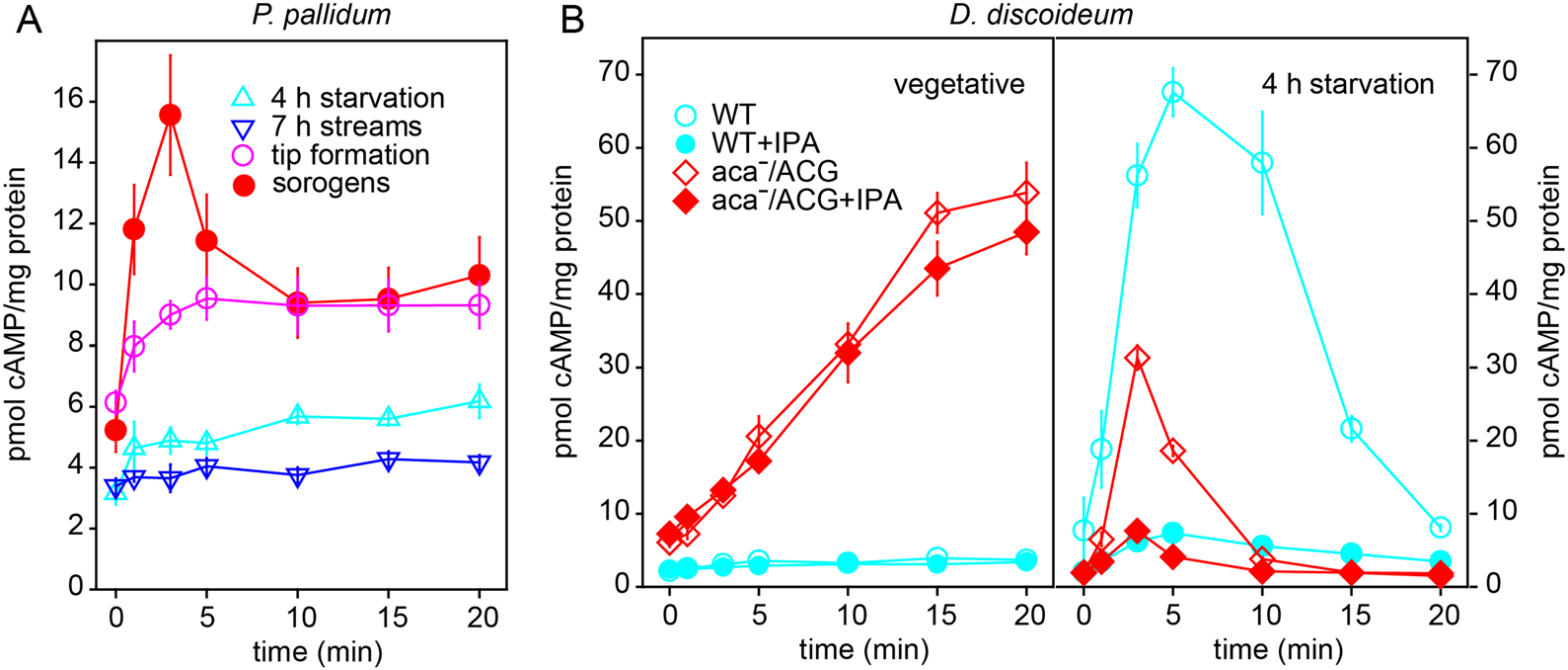
cAMP relay in* Ppal* wild-type and *Ddis acaˉ/ACG. ****A**** P. pallidum. Ppal WT* was starved on agar for 4 h or until streaming aggregates, tipped mounds and aerially lifted sorogens had formed. Structures were gently dissociated, resuspended in PB to 10^8^ cells/ml and stimulated at t = 0 min with 10 μM 2'H-cAMP and 5 mM DTT. Reactions were terminated with 1.75% perchloric acid at the indicated timepoints and cAMP was assayed by isotope dilution assay. ***B*** *D. discoideum* Vegetative WT AX3 and *acaˉ/ACG* cells, and cells starved on NN agar for 4 h were resuspended in PB to 10^8^ cells/ml and stimulated with 5 μM 2'H-cAMP and 5 mM DTT in the presence and absence of 1 mM IPA. Reactions were terminated as above, and cAMP was assayed. Data were standardized to the protein content of the cell suspensions and represent means and SE of six experiments performed in triplicate for *Ppal* sorogens and two experiments in triplicate for other stages and cell lines. The experiments in panel B were performed twice more in triplicate with *acaˉ/ACG* cells in the absence of IPA, with similar results

To investigate whether *Ppal* also shows transient cAMP-induced cAR mediated accumulation of cAMP, we stimulated wild-type *Ppal* cells at different stages of development with the cAR agonist 2'H-cAMP in the presence of the PdsA inhibitor DTT [26]. Figure 5A shows that cells at all stages contain a basal level of 3–6 pmol cAMP/mg protein. Starving cells or cells from streaming aggregates showed none or marginal responses to 2'H-cAMP, while cells from tipped mounds showed a 5 pmol/mg protein increase in cAMP, which then levelled off. However, cells from dissociated sorogens showed transient increase that peaked after 3 min after stimulation at 11 pmol above basal levels and then decreased to 5 pmol. These data indicate that *Ppal* can relay a pulse of cAMP, but only after tips and sorogens have formed. In *Ddis*, which unlike *Ppal* also uses cAMP to aggregate, cAMP relay is highest at the aggregation stage [27]. We could not meaningfully measure 2'H-cAMP-induced cAMP accumulation in the *aca1ˉaca3ˉaca2ˉ* cells, because only few aggregates are formed at different times, which then fragment and fairly rapidly mature into fruiting bodies. This means that at any time only a very small fraction of cells is in the sorogen stage.

Apart from Aca1, Aca2 and Aca3, two other adenylate cyclases, AcgA and AcrA are expressed in *Ppal* sorogens [28]. The experiment in Fig. 5A does not identify the adenylate cyclase responsible for the cAMP increase. While currently not feasible in *Ppal*, a *Ddis acaA* knock-out is available that expresses *AcgA* (ACG) from the constitutive actin 15 promoter [29]. During growth, this mutant synthesizes cAMP at a constant rate [30], but it is unknown whether cAMP synthesis comes under cAR regulation at a later stage. We compared 2'H-cAMP-induced cAMP accumulation between *Ddis* wild-type and *acaˉ/ACG* cells in vegetative and 4 h starved cells, which are just starting to aggregate. To validate that the observed responses are mediated by *Ddis* cAR1, we included the cAR1 antagonist 2′3′-O-isopropylidene adenosine (IPA) in control assays. Figure 5B shows that wild-type *Ddis* shows no 2'H-cAMP-induced cAMP accumulation in the vegetative stage and a 70 pmol/mg protein increase in 4 h starved cells that peaks at 5 min. This response is almost completely inhibited by IPA. Vegetative acaˉ/ACG cells show a steady increase in cAMP levels after addition of 2'H-cAMP/DTT that is only slightly reduced in the presence of IPA. However, 4 h starved acaˉ/ACG cells show a faster transient increase of cAMP that peaks at 3 min after 2′H-cAMP/DTT stimulation. This response is also strongly reduced by IPA. These data indicate that in early aggregating *Ddis* cells, ACG is also controlled by cAR stimulation. The apparent ability of other adenylate cyclases than ACA to participate in transient cAR mediated cAMP accumulation provides some resolution for the contrasting effects on *Ppal* fruiting body morphogenesis of *aca* deletion on one hand, and *cAR* or *pdsA* deletion on the other*.*

## Discussion

### Gene amplification of *aca* genes and their expression in *P. pallidum*

Representative species of the *Dictyostelium* taxon groups 1, 3 and 4 have a single gene each of the adenylate cyclases *acaA*, *acrA* and *acgA*, but in taxon group 2, the ancestral *acaA* gene was amplified twice in *Ppal* and three times in *A. subglobosum* (Additional File 1, Fig. S1). In *Ddis*, a signaling network that critically incorporates ACA, cAR1 and PdsA generates the cAMP pulses that coordinate aggregation and cell movement in the multicellular stage [24, 29]. Deletion of the two *Ppal car* genes, *carA* and *carB,* or its single *pdsA* gene had no effect on aggregation but disorganized the subsequent formation of sorogens and fruiting bodies [11, 12]. This suggested that similar to *Ddis*, *Ppal* multicellular morphogenesis is organized by cAMP pulses. The *Ppal* attractant for aggregation is likely the dipeptide glorin, since starving *Ppal* cells chemotactically respond to glorin [9] and their aggregation is disrupted by including glorin in the supporting agar [5].

We here show that *Ppal aca1* was poorly expressed and only visible in the tips of primary and secondary sorogens, while *aca2* and *aca3* expression was already visible in aggregates. Both genes were preferentially expressed in tip and stalk cells, but *aca2* was also expressed in prespore cells. The latter expression likely explains why double loss of *acrA* and *acgA*, which in *Ddis* leads to complete loss of prespore and spore differentiation [31], only mildly affects *Ppal* sporulation [20]. The post-aggregative expression pattern of either *Ppal aca* resembles that of *Ddis acaA*, which is also preferentially expressed in the organizing tip, from which the organizing cAMP waves emanate [4, 15, 16].

### *Ppal aca3* is required for PKA activation and early development

Deletion of individual *Ppal aca* genes caused subtle developmental defects (summarized in Table 1), with *aca1ˉ* displaying longer, thinner stalks, *aca2ˉ* delayed separation of the first whorl from the main sorogen and *aca3ˉ* showing delayed and reduced formation of aggregation centres, giving rise to extensive streaming (Fig. 2). Double *aca1ˉaca2ˉ* and *aca1ˉaca3ˉ* knock-outs combined the phenotypes of the individual knock-outs, but *aca3ˉaca2ˉ* was very delayed in aggregation and like *aca1ˉaca3ˉaca2ˉ* only formed a few aggregates on an entire plate of cells. The latter mutant did, however, form small fruiting bodies with some whorls of side branches near the top after a very long delay (Fig. 3). Both the delayed aggregation and delayed secondary sorogen formation are non-cell autonomous defects as they are restored by chimeric development of the mutants with 10% wild-type cells.

**Table 1:** Phenotypes of *Ppal aca* knock-outs

Mutant	Phenotype	
	Aggregation	Morphogenesis
*aca1ˉ*	Normal	Somewhat thinner and longer stalks
*aca2ˉ*	Normal	Delayed whorl separation
*aca3ˉ*	Delayed, few centers, long streams	Normal
*aca1ˉaca2ˉ*	Normal	Long whorl-free lower stalk, thin tall fruiting bodies
*aca1ˉaca3ˉ*	Delayed, few centers, long streams	Somewhat thinner and longer stalks
*aca3ˉaca2ˉ*	Very delayed, few centres, long streams	Long whorl-free lower stalk
*aca1ˉaca3ˉaca2ˉ*	Very delayed, very few centres, long streams	Thin tall fruiting bodies, whorls only near top

The delay in aggregation caused by loss of *aca3* was somewhat enigmatic, since no such delay occurred in *Ppal carAˉcarBˉ* or *pdsAˉ* mutants, which cannot detect or hydrolyse extracellular cAMP, respectively. Exposure of the *aca1ˉaca3ˉaca2ˉ* mutant to the membrane-permeant PKA agonist 8Br-cAMP restored normal aggregation (Fig. 4) indicating that Aca3 provides cAMP for activation of PKA in early development. Because defective aggregation of *aca3ˉ* is also restored by wild-type cells*,* this likely means that PKA induces genes required for glorin synthesis. In early *Ddis* development PKA also acts to induce expression of aggregation genes [19].

### The *Ppal aca* genes are not essential for post-aggregative morphogenesis

Despite its long delay in forming aggregates *Ppal aca1ˉaca3ˉaca2ˉ* cells were still able to form relatively normal fruiting bodies with stalk and spore cells. In view of observations that *Ppal carAˉcarBˉ* or *pdsAˉ* cells are highly defective in fruiting body morphogenesis, this suggests that perhaps a static gradient of cAMP produced by ACR or ACG is sufficient to organize morphogenesis or that either or both of these adenylate cyclases can also participate in an oscillatory network. When expressed from the constitutive A15 promoter in an *acaˉ* background, *Ddis* ACG displays a fairly high level of constitutive activity in the growth stage that is activated by high osmolarity [32]. In wild-type *Ddis*, ACG has an overlapping role with ACR in induction of spore formation and inhibition of spore germination [31, 33]. In *Ppal* ACG and ACR critically regulate encystation, but their role in sporulation is less pronounced [20], which may be due to the additional activity of Aca1, 2 and 3 in sorogens.

### ACG mediates cAMP stimulated transient cAMP accumulation

*Ppal* cells at the sorogen stage show transient 2′HcAMP-induced cAMP synthesis (Fig. 5A), consolidating evidence from *Ppal carˉ* and *pdsAˉ* mutants that cAMP pulses coordinate postaggregative morphogenesis [11, 12], but the *Ppal aca1ˉaca3ˉaca2ˉ* mutant still makes fruiting bodies, suggesting that this is not the case. To resolve this conundrum, we explored whether other adenylate cyclases might mediate pulsatile signaling. We found that when aggregation competent *Ddis acaˉ/ACG* cells are stimulated with 2′HcAMP, ACG mediates a transient accumulation of cAMP (Fig. 5B). Like ACA mediated transient cAMP synthesis, the response is inhibited by the cAR antagonist IPA, indicating that it is mediated by cARs. The experiment shows that at least one of the other dictyostelid adenylate cyclases could also give rise to oscillatory cAMP signalling, which provides some resolution to the conundrum.

**Fig. 6:**
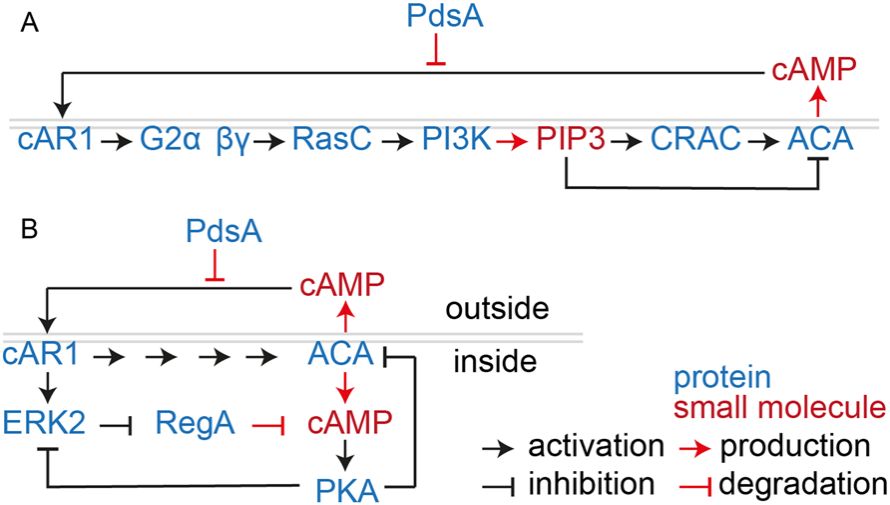
Proposed models for ACA mediated oscillatory cAMP signalling See main text for explanation. Abbreviations: *PdsA* phosphodiesterase A, *cAR1* cAMP receptor 1, *G2*: heterotrimeric G-protein 2, *RasC* small GTPase C, *PI3K*: phosphoinositide 3-kinase, *PIP3* phosphatidylinositol-(3,4,5)-trisphosphate, *CRAC* cytosolic regulator of adenylate cyclase, *ACA* adenylate cyclase A, *ERK2* extracellular signal-regulated kinase 2, *RegA* cAMP phosphodiesterase with response regulator, *PKA* cAMP dependent protein kinase. Schematics are based on Fig. 6 in [35] and Fig. 2 in [36]

How the apparent transient ACG activation occurs is unresolved. Oscillatory cAMP signalling depends on both positive and negative feedback loops acting on cAMP production (Fig. 6). ACA activation involves positive feedback loop, where cAMP synthesized by ACA process binds to cAR1 and initiates a multistep process that causes activation of ACA (see [34]. Negative feedback on ACA may involve PIP3, one of the activating intermediates, also causing inhibition of ACA after a delay [35]. In an alternative model (Fig. 6B), the positive feedback loop involves both cAMP activation of ACA, as above, and of the protein kinase ERK2, which next inactivates the intracellular cAMP phosphodiesterase RegA, enabling cAMP to increase. The negative loop involves PKA activation by cAMP synthesized by ACA, inactivation of ERK2 and thereby activation of RegA [36]. In this model it is mostly cAMP degradation that is under positive and negative feedback regulation, making it conceivable that a constitutively active adenylate cyclase-like ACG or ACR could still display apparent transient activation.

## Conclusions

In *Ddis,* cAMP waves produced by ACA and emanating from aggregation centres and organizing tips control both aggregation and post-aggregative morphogenesis. We here investigated ACA function in *Ppal*, which likely uses glorin for aggregation. *Ppal* has 3 *aca* genes, which similar to *Ddis aca* are most highly expressed in the organizing tip. Deletion of either gene causes different but relatively subtle changes in aggregation and post-aggregative morphogenesis, while deletion of all three *aca* genes causes a long delay in aggregation with only few centres being formed that attracted long streams of amoebas. The fragmenting streams eventually gave rise to small fruiting bodies. Timely aggregation was restored by including the PKA agonist 8Br-cAMP in the substratum, indicating that in early development the *Ppal* ACAs are needed to activate PKA.

While the formation of fruiting bodies by the *Ppal aca1ˉaca3ˉaca2ˉ* mutant argues against a role for ACAs and thereby oscillatory cAMP signalling in fruiting body morphogenesis, several lines of evidence indicate the opposite. 1. Loss of other components that are essential for cAMP oscillations, such as cARs or pdsA disorganizes morphogenesis. 2. Post-aggregative *Ppal* cells produce a cAMP pulse when stimulated with 2′H-cAMP, a cAMP receptor agonist. 3. In *Ddis* other adenylate cyclases, such as ACG also mediate transient 2′H-cAMP-induced cAMP accumulation, at a stage when cARs and PdsA are also present.

Overlapping roles of ACG and ACR were detected in *Ddis* sporulation [31] and *Ppal* encystation, while the *Ppal* ACAs were proposed to overlap with ACG and ACR in induction of sporulation [20]. The current study provides hints that ACG and possibly ACR in turn overlap with ACA in morphogenetic signalling. Altogether, ACA, ACG and ACR may ancestrally have been less specialized and acquired their specific roles in the course of dictyostelid evolution by expression in different cell types and interaction with proteins specific to that cell type.

## Methods

### Growth and development.

*P. pallidum* PN500 (*Ppal*) was routinely grown in association with *Escherichia coli* on LP agar or 1/5^th^ SM agar (Formedium, UK). For multicellular development, cells were harvested in 20 mM K-phosphate, pH 6.5 (KK2), washed free from bacteria and incubated at 10^6^ cells/cm^2^ and 22 °C on NN agar (1.5% agar in 8.8 mM KH_2_PO_4_ and 2.7 mM Na_2_HPO_4_) until the desired developmental stages had been reached. *D. discoideum* (*Ddis)* AX3 and *acaˉ/ACG* cells [29] were grown in HL5 axenic medium, which was supplemented with 20 μg/ml G418 for *acaˉ/ACG.*

### DNA constructs and transformation

#### Ppal Aca promoter-lacZ constructs and analysis

To construct a gene fusion of the *Ppal aca1* promoter and *lacZ*, an *aca1* (PPL_01657) fragment 3511 nt upstream and 93 nt downstream of the start ATG was amplified from *Ppal* gDNA using primers Pp-ACA1-P52X with *Xba*I site and Pp-ACA1-P32. The fragment was digested with *Xba*I and *Bam*HI (using an internal *Bam*HI site) and ligated into the *Bgl*II/*Xba*I digested pDdGal17 [37], yielding pPpACA1-LacZ. The *aca2* (PPL_12370) 3.8 kb 5'intergenic region (− 3743 to + 86) was amplified using primers Pp-ACA2-P51E and Pp-ACA2-P31B that harbour *Eco*RI and *Bam*HI, respectively. The *Eco*RI/*Bam*HI digested PCR product was ligated into the *Eco*RI/*Bgl*II digested pDdGal16 [37], yielding vector pPpACA2-LacZ. The *aca3* (PPL_10658) 2.6 kb 5′ intergenic region (− 2502 to + 50) was amplified using primers Pp-ACA3-P52X and Pp-ACA3-P32B that harbour *Xba*I and *Bam*HI, respectively. The *Xba*I/*Bam*HI digested PCR product was ligated into similarly digested pDdGal17, yielding vector pPpACA3-LacZ. After validation of the plasmids by DNA sequencing, they were transformed into *Ppal* wild-type cells. Transformants were selected at 300 µg/ml G418 [38] and pools of 7–10 transformed clones were developed into multicellular structures on dialysis membrane, supported by NN agar. β-galactosidase activity was visualized with X-gal in the structures, as described previously [11, 39].

#### Ppal gene knock-out constructs

To disrupt *Ppal aca1* (PPL_01657), an *aca1* fragment was amplified from *Ppal* PN500 genomic DNA using primers Pp-ACA-51H and Pp-ACA-31B (Additional file 1: Table S1) that harbour *Hind*III and *Bam*HI restriction sites, respectively. The fragment was cloned into *Bam*HI/*Hind*III digested pBluescript SK +  which was next digested with EcoRV. The LoxP-NeoR cassette of pLoxNeoIII [12] was excised with BamHI and HindIII, filled in with Klenow polymerase, and ligated into the EcoRV digested *aca1* plasmid, yielding vectors pACA1-KO1 and pACA1-KO2, with loxP-NeoR inserted in *aca1* in forward and reverse orientation, respectively, and flanked by 2307 bp 5′UTR and 5’*aca1* sequence and 1414 bp 3’*aca1* sequence (Additional file 1: Fig. S2A). pACA1-KO2 was used for gene disruption.

To disrupt *Ppal aca2* (PPL_12370)*,* an *aca2* fragment was amplified using primers Pp-ACA2-51 K and Pp-ACA2-31S (Additional file 1: Table S1) that harbour *Kpn*I and *Sac*I sites, respectively. The fragment was cloned into *Kpn*I/*Sac*I digested pBluescript SK +  which was next digested with *Bam*HI/*Sal*I. LoxP-NeoR was excised with *Bam*HI/*Sal*I from pLoxNeoIII and ligated into the *Bam*HI/*Sal*I digested *aca2* plasmid, yielding vector pACA2-KO with LoxP-NeoR flanked by 1918 bp 5’*aca2* sequence and 3086 bp 3′*aca2* and 3′UTR sequence (Additional file 1: Fig. S2B).

To disrupt *Ppal aca3* (PPL_10658), two *aca3* sequences, A and B, were amplified using primer pair Pp-ACA3-51 K/Pp-ACA3-31X, that harbour *Kpn*I and *Xba*I sites for A and primer pair Pp-ACA3-52B/Pp-ACA3-32X with *Bam*HI and *Xba*I sites for B, respectively. Fragment A was digested with *Kpn*I/*Sal*I (using an internal *Sal*I site) and inserted into *Kpn*I/*Sal*I digested pLox-NeoIII, and next *Bam*HI/*Xba*I digested fragment B was inserted into the *Bam*HI/*Xb*aI sites of the resulting vector, yielding pACA3-KO with 2042 bp 5′ *aca3* sequence and 2531 bp 3’ *aca3* and 3’UTR sequence. (Additional file 1: Fig. S2C).

*Ppal* cells were transformed by electroporation with the linearized vectors according to established procedures [38]. Genomic DNA was isolated from G418 resistant clones and analyzed by PCR and Southern blots to diagnose gene disruption by homologous recombination (Additional file 1: Fig. S2).

To generate double *aca1ˉaca2ˉ* or *aca1ˉaca3ˉ* knock-outs, the loxP-NeoR cassette was removed from *aca1ˉ* by transient transformation with pA15NLS.Cre [17]. Cells that had regained sensitivity to G418 were then transformed with the pACA2-KO or pACA3-KO plasmids. This strategy did not work for the *aca2ˉ* and *aca3ˉ* knockouts. To generate an *aca3ˉaca2ˉ* double and *aca1ˉaca3ˉaca2ˉ* triple knockout, *aca3ˉ* and *aca1ˉaca3ˉ* were transformed with pDM1483 [18], which harbours cassettes for Nourseothricin selection and cre-recombinase expression. Transformants were selected after growth for 3–5 days in the presence of 300 µg/ml Nourseothricin, and, after replica-plating, selected for G418 sensitivity and transformed with the pACA2-KO vector. All gene knock-outs were diagnosed by PCR and/or Southern blot (Additional file 1: S2).

### cAMP relay assays

To measure cAMP-induced cAMP accumulation, *Ppal* cells were resuspended in PB (10 mM Na/K-phosphate, pH 6.5) at 10^8^ cells/ml, dispensed as 25 μl aliquots in microplate wells and, stimulated with 5 μl of 60 μM 2'H-cAMP (2′-deoxyadenosine 3′:5′-cyclic monophosphate, Sigma-Aldrich, in 30 mM DTT (dithiothreitol, Sigma-Aldrich) and shaken at 100 rpm and 21 °C. Reactions were terminated by addition of 30 μl of 3.5% (v/v) perchloric acid. *Ddis* cells additionally received 3 μl of 10 mm IPA (2′,3′-O-isopropylideneadenosine, Sigma-Aldrich) in 10% (v/v) (DMSO) dimethylsulfoxide, Sigma-Aldrich) or 3 μl 10% DMSO (controls) and were stimulated with 3 μl 50 μM 2′H-cAMP in 50 mM DTT. For cAMP assay, samples were neutralized by addition of 15 μl of 50% saturated KHCO_3_ and 75 μl of cAMP assay buffer (4 mM EDTA in 150 mM K phosphate, pH 7.5). Protein and perchlorate were precipitated by centrifugation for 5 min at 2000 ×g and cAMP was assayed in 50 μl of supernatant by isotope dilution assay using purified PKA regulatory subunit from beef heart as cAMP-binding protein and [2,8-^3^H]cAMP (Perkin–Elmer) as competitor [26, 40].

## Supplementary Information

**Additional file 1:** **Figure S1.** *acaA* genes across Dictyostelia. **Figure S2.** Schematics and diagnosis of *Ppal aca1*, *aca2* and *aca3* knock-outs. **Figure S3.** Encystation. **Table S1.** Oligonucleotide primers used in this work.

## Data Availability

All data generated or analyzed during this study are included in this article and Additional file 1. The DNA constructs and knock-out cell lines produced in the study will be deposited in the *Dictyostelium* Stock Centre http://dictybase.org/StockCenter/StockCenter.html
