# Supplementary material for: Adenylate cyclase A amplification and functional diversification during Polyspondylium pallidum development
Source: EvoDevo. 2022 Oct 19;13:18. doi: 10.1186/s13227-022-00203-7 (PMC9583560; doi:10.1186/s13227-022-00203-7)
Supplement: Supplementary file 1 — Additional file 1: Figure S1. acaA genes across Dictyostelia. Figure S2. Schematics and diagnosis of Ppal aca1, aca2 and aca3 knock-outs. Figure S3. Encystation. Table S1. Oligonucleotide primers used in this work. [file 13227_2022_203_MOESM1_ESM.docx]

**ADDITIONAL FILE 1**

Additional figures S1-S3, additional table S1.

**
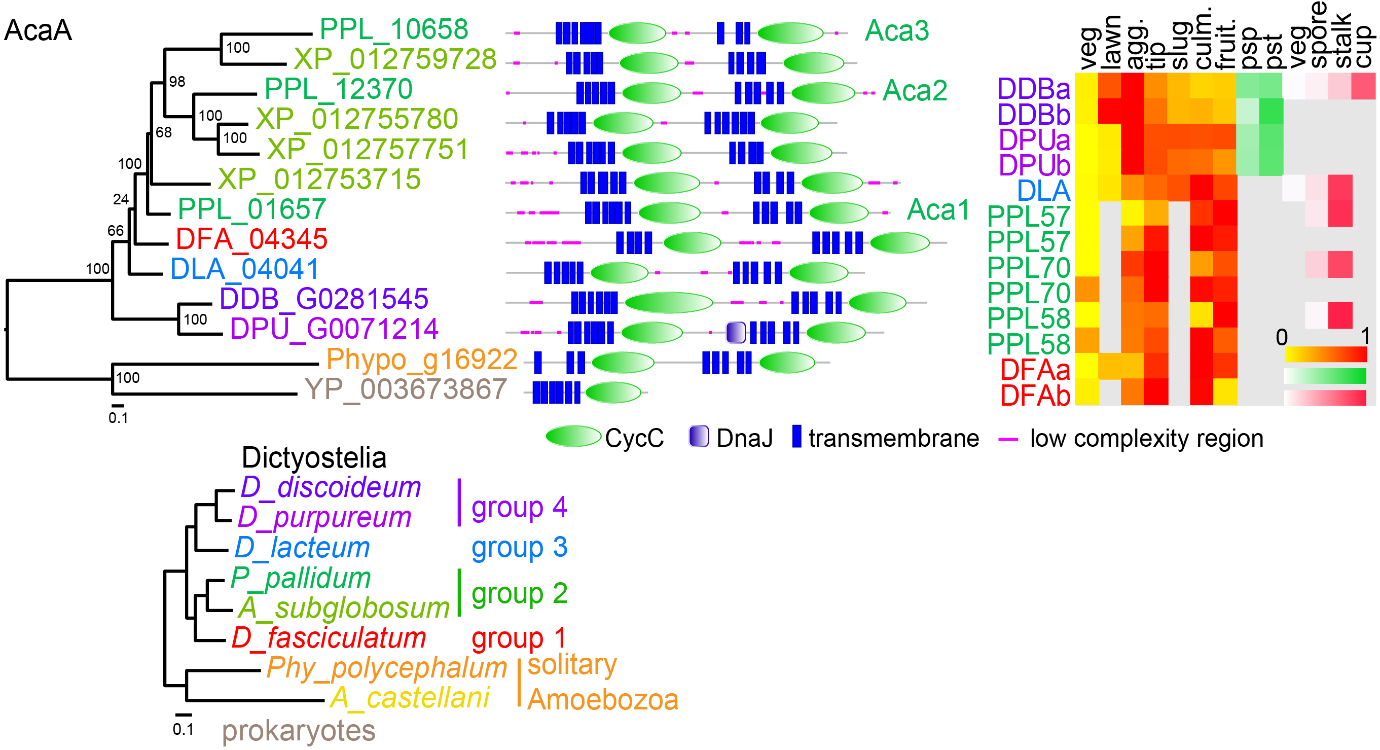
**

[**Additional File 1**](sps:refid::MOESM1)**: Figure S1. *acaA* genes across Dictyostelia**

*Ddis* AcaA homologs were retrieved by BlastP from genomes of taxon group representative Dictyostelia, some unicellular Amoebozoa and the top hit outside of Amoebozoa. After sequence alignment, a phylogenetic tree (top image) was inferred from aligned sequences using IQtree [1]. Percentage bootstrap support is shown at the nodes and the scale bar reflects the number of amino acid substitutions per site. Gene names are colour coded to reflect the host species as shown in the multigene phylogeny of Dictyostelia (derived in [2]). The tree was annotated with the domain architectures of the proteins as analysed in SMART [3]. CycC: Adenylyl-/guanylyl cyclase catalytic domain, DnaJ: molecular chaperone homology domain. Heatmaps are shown of relative transcript levels at different developmental stages (yellow-red: 0-1 fraction of the maximum reads per individual time course), prespore or prestalk cells (white-green: 0-1 fraction of summed reads), or vegetative, spore, stalk and cup cells (white-red: 0-1 fraction of summed reads). Normalized transcript reads for developmental time courses were retrieved for *Ddis* (DDBa, DDBb) and *D. purpureum* (DPUa, DPUb) from a replicate experiment [4] and for *D. fasciculatum* (DFA) and *Ppal* from two separate experiments, using either the Roche 454 (DFAa, PPLa) or the Illumina sequencing platforms (DFAb, PPLb) [5, 6]. The three entries for the Ppal ACAs further coded by the last two digits of their locus tags. For *D. lacteum* (DLA) a single time course RNAse experiment was available [6]. Additionally, the average of a triplicate RNAseq experiment of purified *Ddis* vegetative, spore, stalk and cup cells [7] is shown with the DDBa time course and a single experiment of purified *D. lacteum* vegetative. stalk and spore cells with the DLA time course [8].

**
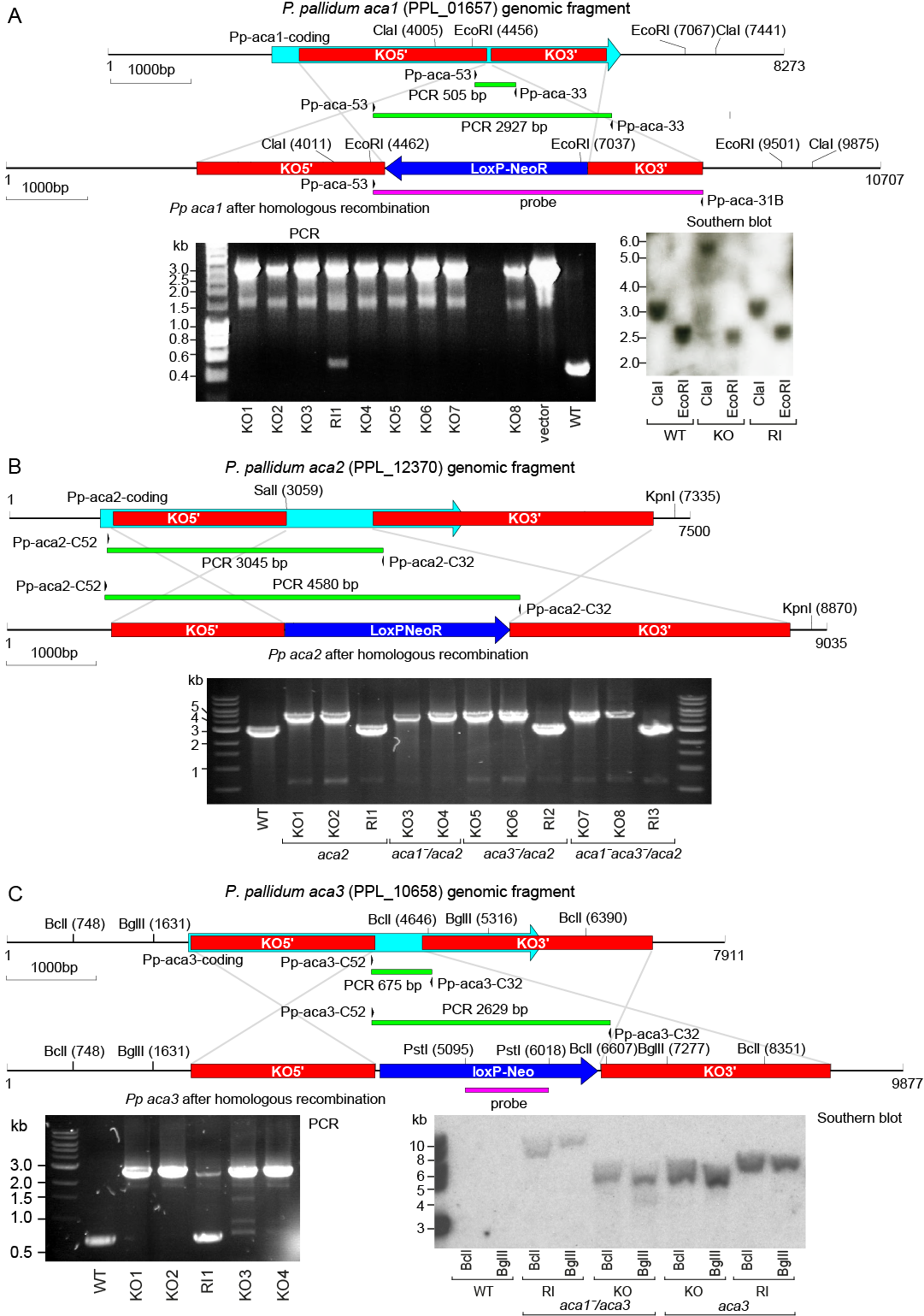
**

[**Additional File 1**](sps:refid::MOESM1)**: Figure S2. Schematics and diagnosis of *Ppal aca1*, *aca2* and *aca3* knock-outs**

A. *Ppal aca1*. *Top:* Schematic of the *Ppal aca1* (PPL_01657) genomic region before and after homologous recombination with knock-out vector pACA1-KO2. The location of primers and relevant PCR products, restriction sites and probe for southern blot analysis are indicated. *Bottom left:* PCR amplification of gDNAs of 8 knock-out (KO) clones, one random integrant (RI) clone, wild-type cells (WT) and vector DNA with primers Pp-aca-53 and Pp-aca-33. From WT and RI gDNA, the expected 505 bp bands were amplified, while only 2927 bp bands were amplified in KO clones. *Bottom right:* WT, KO and RI gDNAs were digested with ClaI or EcoRI and Southern blots were probed with the indicated ^32^PdATP-labeled fragment. In WT and RI, the expected 3.4 kb (ClaI digested) and 2.6 kb (EcoRI digested) band were detected, while the KO ClaI and EcoRI digests showed the expected 5.8 kb and 2.5 kb bands, respectively.

*B. Ppal aca2. Top:* Schematic of the *Ppal aca2* (PPL_12370) genomic region before and after recombination with vector pACA2-KO, with the location of the primers used to diagnose recombination. *Bottom:* PCR diagnosis of *aca2* knock-out in WT, *aca1ˉ* and *aca1ˉaca3ˉ Ppal.* from WT and RI gDNAs tShe expected 3 kb bands were amplified, while 4.6 kb products were amplified from KO gDNAs. All tested RI clones showed same phenotypes as their parental strains.

*C.* *Ppal aca3. Top:* Schematic of the *Ppal aca3* (PPL_10658) genomic region before and after recombination with vector pACA3-KO. The location of primers and relevant PCR products, restriction sites and probe for southern blot analysis are indicated. *Bottom left:* PCR amplification of gDNAs of KO, RI and WT cells. In the WT and RI, a 675 bp band was amplified, while a 2.6 kb band was amplified in 4 KO clones

*Bottom right:* gDNAs of a WT and a KO and RI clone each of WT and *aca1ˉ* cells transformed with pACA3-KO were digested with *Bcl*I *or Bgl*II. Southern blots were probed with a ^32^P-dATP-labeled 0.9 kb internal PstI fragment of the LoxP-Neo cassette. In the KO mutants, ~6 kb bands (*Bcl*I digested) and ~5.5 kb (*Bgl*II digested) band were detected, similar to the expected sizes (5859 bp and 5646 bp respectively).

**
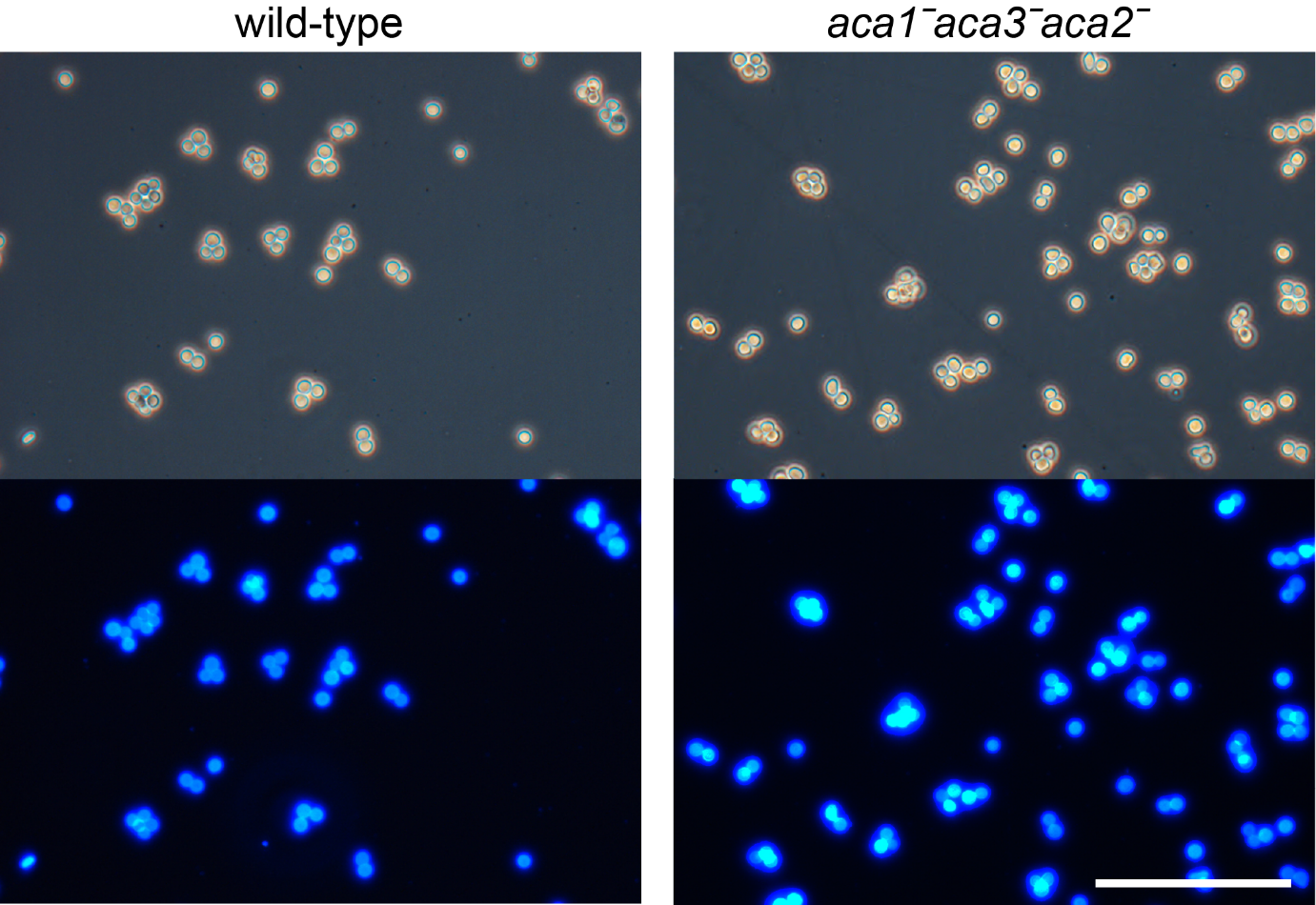
**

[**Additional File 1**](sps:refid::MOESM1)**: Figure S3. Encystation**

Wild type and *aca1ˉaca3ˉaca2ˉ* cells were incubated at 10^7^ cells/ml in 0.2 M sorbitol in KK2 for 24 hr at 22°C. The cells were stained stained with 0.001% Calcofluor and photographed under phase contrast (top) and epifluorescence (bottom). Scale bar: 20 µm.

**[Additional File 1](sps:refid::MOESM1): Table S1. Oligonucleotide primers used in this work**

| promoter-*lacZ* constructs | |
| --- | --- |
| Pp-ACA1-P52X | GCTCTAGAGTTGTAAGATATGTACATCTACCGGCATCAG |
| Pp-ACA1-P32 | CTGCTGAGAACATTGCTACTGCTACTATTATG |
| Pp-ACA2-P51E | GGAATTCGAGGAAATCACGATAGCTTAAAAACTCTG |
| Pp-ACA2-P31B | CGGGATCCGTTATGGGTGAATTGTGACTATTTGAGGTG |
| Pp-ACA3-P52X | GCTCTAGATGACGATGATGCTAATGAACAGCAAGAGGA |
| Pp-ACA3-P32B | CGGGATCCTCACGATTCGCAGTAAAACTTGCTCCATAG |
| knock-out constructs and diagnoses | |
| Pp-ACA-51H | CCCAAGCTTGTGTAAGCTCACCGCCAACCT |
| Pp-ACA-31B | CGGGATCCGCTGTCGGTTGAACCTCGCTT |
| Pp-ACA-53 | ACAACCGGTGAAAAGTCAAC |
| Pp-ACA-33 | TGATGAAAGACATGGCGAAA |
| Pp-ACA2-51K | GGGGTACCGCCGACAAACGCAAAAGGAC |
| Pp-ACA2-31S | CGAGCTCCAAGACTCGGCGATACCGTAA |
| Pp-ACA2-C52 | CCCATAACACCTCGAACT |
| Pp-ACA2-C32 | CAAATTCTTCGCATTCCCA |
| Pp-ACA3-51K | GGGGTACCGGAGCAAGTTTTACTGCGAA |
| Pp-ACA3-31X | GCTCTAGACATGTCCTGAATCGATTGTCTG |
| Pp-ACA3-52B | CGGGATCCTACACACCAGATTGGATCGT |
| Pp-ACA3-32X | GCTCTAGAACTGCTGTCTTTGTGACTC |
| Pp-ACA3-C52 | TGTTATCCGGCGCTATCGTG |
| Pp-ACA3-C32 | GCTGAAAGTGGTGGCGTTTG |

**ADDITIONAL REFERENCES**

1. Trifinopoulos J, Nguyen L-T, von Haeseler A, Minh BQ: W-IQ-TREE: a fast online phylogenetic tool for maximum likelihood analysis. Nucleic Acids Research 2016; 44(W1):W232-W235.

2. Singh R, Schilde C, Schaap P: A core phylogeny of Dictyostelia inferred from genomes representative of the eight major and minor taxonomic divisions of the group. BMC Evol Biol 2016; 16(1):251.

3. Schultz J, Milpetz F, Bork P, Ponting CP: SMART, a simple modular architecture research tool: identification of signaling domains. Proc Natl Acad Sci USA 1998; 95(11):5857-5864.

4. Parikh A, Miranda ER, Katoh-Kurasawa M, Fuller D, Rot G, Zagar L, Curk T, Sucgang R, Chen R, Zupan B *et al*: Conserved developmental transcriptomes in evolutionarily divergent species. Genome Biol 2010; 11(3):R35.

5. Heidel A, Lawal H, Felder M, Schilde C, Helps N, Tunggal B, Rivero F, John U, Schleicher M, Eichinger L *et al*: Phylogeny-wide analysis of social amoeba genomes highlights ancient origins for complex intercellular communication. Genome Res 2011; 21:1882-1891.

6. Gloeckner G, Lawal HM, Felder M, Singh R, Singer G, Weijer CJ, Schaap P: The multicellularity genes of dictyostelid social amoebas. Nature communications 2016; 7:12085.

7. Kin K, Forbes G, Cassidy A, Schaap P: Cell-type specific RNA-Seq reveals novel roles and regulatory programs for terminally differentiated Dictyostelium cells. BMC Genomics 2018; 19(1):764.

8. Forbes G, Chen ZH, Kin K, Lawal HM, Schilde C, Yamada Y, Schaap P: Phylogeny-wide conservation and change in developmental expression, cell-type specificity and functional domains of the transcriptional regulators of social amoebas. BMC Genomics 2019; 20(1):890.
